# Enhancing Picture Naming with Transcranial Magnetic Stimulation

**DOI:** 10.1155/2006/768413

**Published:** 2006-11-27

**Authors:** Felix M. Mottaghy, Roland Sparing, Rudolf Töpper

**Affiliations:** ^1^Department of Nuclear MedicineUniversity Hospital UlmRobert-Koch-Str. 889081 UlmGermany; ^2^Department of NeurologyRWTH AachenPauwelsstr. 3052074 AachenGermany; ^3^Department of NeurologyAK Hamburg-HarburgEissendorfer Pferdeweg 52 21075 HamburgGermany

## Abstract

The enhancement of cognitive function in healthy subjects by medication, training or intervention yields increasing political, social and ethical attention. In this paper facilitatory effects of single-pulse TMS and repetitive TMS on a simple picture naming task are presented. A significant shortening of picture naming latencies was observed after single-pulse TMS over Wernicke's area. The accuracy of the response was not affected by this speed effect. After TMS over the dominant motor cortex or over the non-dominant temporal lobe, however, no facilitation of picture naming was observed. In the rTMS experiments only rTMS of Wernicke’s area had an impact on picture naming latencies resulting in a shortening of naming latencies without affecting the accuracy of the response. rTMS over the visual cortex, Broca's area or over the corresponding sites in the non-dominant hemisphere had no effect. Single-pulse TMS is able to facilitate lexical processes due to a general preactivation of language-related neuronal networks when delivered over Wernicke's area. Repetitive transcranial magnetic stimulation over Wernicke’s area also leads to a brief facilitation of picture naming possibly by shortening linguistic processing time. Whether TMS or rTMS can be used to aid linguistic therapy in the rehabilitation phase of aphasic patients should be subject of further investigations.

## 1. Introduction

A number of recent papers have addressed the potential of transcranial magnetic stimulation TMS to interfere with linguistic processes or speech production [7, 9,17,18,26]. TMS can be used to identify the language dominant hemisphere by targeting the language relevant areas (i.e. temporal, parietal or prefrontal cortex) of both sides. This approach is based on the assumption that TMS can disrupt normal brain function. Another series of experiments studied specific linguistic functions such as verb or noun production, object naming or grammatical decisions etc. These studies, which are primarily addressing the functional localization of language-related networks in the brain, also explored the possibility to enhance or inhibit definite linguistic functions.

The so called virtual lesion approach, i.e. the TMS-induced inhibition of function is meanwhile a well accepted method in cognitive neuroscience. The enhancement of function [3] by TMS or rTMS, however, has received some criticism [33]. Some claim that enhancement of function at least in part might be an unspecific effect of brain stimulation. Critics argue that the improvement of specific functions could be due to intersensory facilitation, unspecific arousal reactions or enhancement of attention [6]. If TMS can improve function this would have an impact in neurorehabilitation of brain-damaged patients. In addition it is intriguing to speculate that TMS could in the future also be used in healthy subjects to enhance function.

It is generally accepted that TMS or rTMS applied to a circumscribed cortical area has not only a local effect but can also influence functionally connected brain regions. TMS therefore seems to be able to modulate areas which are remote from the site of stimulation.

ISSN 0953-4180/06/$17.00 © 2006 – IOS Press and the authors. All rights reserved

In this paper we will review some data on short-term effects of TMS and repetitive TMS in healthy male subjects on a simple object naming task.

The first study inducing speech arrest by high-frequency rTMS in epileptic patients showed a significant correlation to the WADA test [42], suggesting the inauguration ofa new non-invasive tool to test hemispheric laterality [26]. The exact identification of the language-dominant hemisphere is, however, not possible in all subjects [21]. Some researchers claim that in almost all subjects a speech arrest can be achieved by stimulating the prefrontal cortex or the facial motor cortex of the dominant hemisphere [7]. However, it seems that lateralization is less clear using this approach since an unexpectedly high number of subjects also show effects with bilateral or non-dominant hemisphere stimulation [7]. This is possibly due to the fact that in a general population a graded continuum of lateralization from left to right for language functions can be found [18]. In a very elegant approach a clear correlation between the lateralization index as determined by functional transcranial Doppler sonography and rTMS interference with language function (i.e. picture-word verification) has been shown [18]. Interestingly subjects with a more bilateral distribution of language function were less susceptible to rTMS interference.

Following a train of 1-Hz rTMS over the left prefrontal cortex a differentiation of verb and noun processing within the left prefrontal cortex has been shown [36]. In this study it appeared that low-frequency trains, applied over a portion of the left mid-frontal gyrus, had a disproportionately large impact on the retrieval of information relating to the grammatical role of verbs in comparison to the category noun. This is consistent with reports of verb production deficits associated with left frontal lesions in aphasic patients [36].

An evolutionary concept of language that traces the origin of language back to gestures could explain the observation that aphasic right-handed patients can improve object naming by movements of their right upper extremity, i.e. pointing towards the location where the object will be presented or activating the proximal muscles of the upper extremity if the arm is paretic [12, 13]. Several studies demonstrated an increased motor cortical excitability specifically pronounced within the hand motor cortex induced by speech listening underlining the strong link between gesture and language [8, 9]. Strong evidence for the evolutionary link between the motor cortex, gesture and speech was demonstrated in several TMS studies [19,20,35,40]. TMS could identify an increased excitability in the left hand motor cortex during speech production in healthy subjects [20] whereas in patients recovering from aphasia the excitability of the right hemisphere increased during loud reading [19]. One explanation for the effect of speech listening could be a modulation of inhibiting connections between mirror neurons within Broca's area and the primary motor cortex [32].

These observations were the basis for some of our experiments on language functions using TMS [41] and rTMS [23,38]. Initially the experiments were conceptually build upon the idea that enhancement of motor cortex excitability might facilitate language function in a manner analagous to the effects seen in aphasic patients when they moved their right upper extremity.

Additionally we focused on a cortical region known to be involved in language perception, initiation and processing (i.e. Wernicke's area). Our hypothesis was that by interfering with modules of the language network, a modulation of task performance could be achieved. Since pointing towards a presented object seems to be beneficial for aphasic patients, we postulated that TMS or rTMS given prior to the presentation onset could have the potential to facilitate processing within the language network. A simple naming task using black and white line drawings presented sequentially on a computer screen was used to test this hypothesis in a series of two different experimental sequences [23,38,41]. In the first series the chronometry of this postulated naming facilitation effect was tested with single-pulse TMS at varying time-points with respect to picture onset [41], while the second series focused on the immediate effect of repetitive TMS using different intensities and frequencies [23,38].

## 2. Methods

Across the different experiments [23,38,41] the naming task remained basically unchanged. Subjects were instructed to name as fast as possible objects presented on a computer screen. Pictures were presented for 1 s followed by a blank screen interval of 0.5 s. Each recording session lasted approximately 30 minutes (Fig. 1).

Stimuli were taken out of a set standardized for frequency and visual complexity [37]. These pictures show common every day life objects, such as animals, tools etc. Since not all objects are equally familiar to German subjects (e.g. baseball bat) 160 pictures were

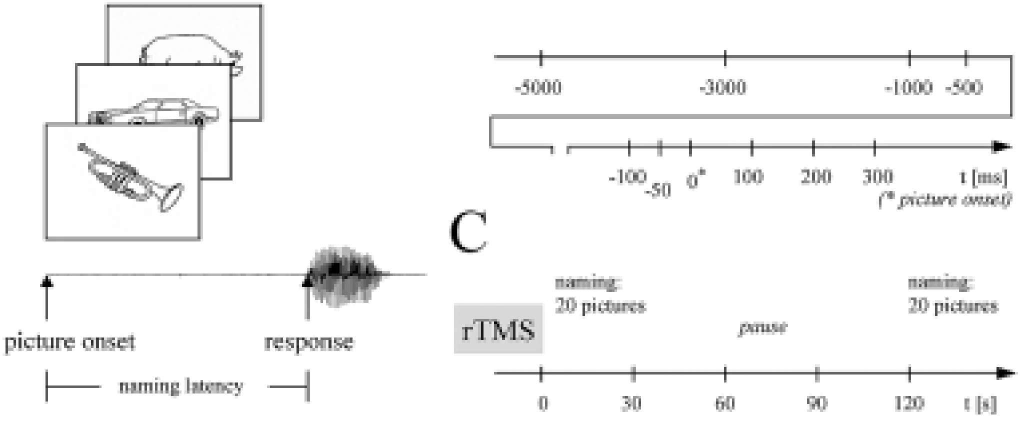

Fig. 1. Experimental design: **A** Black-and-white line drawings [37] were presented to the subjects. Naming latency was defined as the latency between picture onset and the first detectable amplitude in the digitized speech-wave envelopes. **B** In the single pulse-experiments [41] TMS was applied prior to and post picture onset, defined as negative and positive values of the interstimulus interval (ISI), respectively. **C** In the rTMS experiments [23,38] subjects had to name blocks of 20 pictures immediately and 2 min after the end of the high-frequency (20 Hz) or low-frequency (1 Hz) trains.

selected out of the original 240. These were then subdivided into 8 sets of 20 pictures each which were balanced according to familiarity, complexity, semantic content, and syllable length of their word label. In the single-pulse TMS experiments [41] the sequence of pictures within a set was systematically varied. In addition, the sequence of the sets of pictures in a series of experiments was also varied systematically between different subjects in the single-pulse TMS experiments. In the rTMS experiments [23,38] only 10 black and white line drawings of objects, having the smallest standard deviation in naming latency across individuals in the control group, were chosen (further details see below).

Verbal responses of subjects were recorded by a microphone attached to the subject's shirt collar. Acoustic information was digitised at an 8-bit resolution and a sampling rate of 22 kHz and presented as speech wave envelopes. The latency of the verbal responses was measured using the speech wave envelopes. To determine the acoustic onset of the verbal response an amplitude filter was used which removed all acoustic signals with an amplitude of less than 7.5% of the maximum sound level (for a detailed description see [41]).

The semantic correctness of each verbal response was checked off-line. Semantically incorrect responses, as well as responses which were preceded by verbal searching were marked and excluded from further statistical analysis. Verbal searching which is also known as "tip of the tongue phenomenon" can be seen in pauses filled by interjections such as "uh..uh..uh" or by self-commenting phrases as well as by outstandingly prolonged empty pauses. Obviously, such "tip of the tongue phenomena" can have an impact on picture naming latencies and therefore potentially obscure specific facilitatory or inhibitory effects of TMS. Therefore, all responses with "tip of the tongue phenomena" were excluded. Extreme delay was defined as a naming latency which exceeded the control group's average naming latency for a particular picture by more than two standard deviations. Three different categories of exclusions were defined: 1. semantically incorrect responses, 2. responses with multiple onset due to verbal searching and 3. extremely delayed responses. Since TMS could induce semantic errors or be responsible for extreme delays the number of exclusions was calculated and compared between experimental conditions.

The naming latencies of all pictures used in the following experiments were standardized in a first group of 10 subjects (aged between 23 and 29; mean 25.5 years). In all experiments only male native speakers of German were included. None had a history of neurological diseases. All individuals were right-handed according to the Edinburgh handedness inventory [25] and had normal, or corrected to normal, vision. Volunteers gave informed consent to the studies [23,38,41], which were approved by the local institutional review board.

## 3. Data analysis

In order to measure potential facilitatory or inhibitory effects of rTMS, the measured naming latency in a particular experiment was compared to the individual baseline condition of naming without TMS or rTMS. In the single-pulse TMS experiment the standardized naming latency for each picture was additionally taken into account (for a detailed description see [41]). In the rTMS experiment blocks of 20 pictures were presented twice for each stimulation condition as well as for the baseline condition. Mean values were calculated from the pooled data for each condition. To compare between the various stimulation sites and the baseline condition several statistical methods were applied. First an analysis of variance across all conditions was performed ($p < 0.05$). Post-hoc, a Student's paired t-test was performed. The level of significance was set at $p < 0.05$. In addition the nonparametric Friedman two-way rank test was carried out to look for a significance in the mean value of the single conditions ($p < 0.05$). In the Wilcoxon matched-pairs signed rank test the effect of the mean value of single subjects for each conditions ($p < 0.05$) was explored. Finally the data of the rTMS experiment were tested for sequence effects, i.e. a correlation between the order of stimuli and naming latency across the sequence of the presented pictures (Pearson correlation).

## 4. Single pulse TMS in picture naming

In order to study the influence of single-pulse TMS on picture naming latencies the left motor cortex or Wernicke‘s area (BA 22) were stimulated. Different time intervals between picture onset and TMS application were chosen to investigate the chronometry of a potential facilitatory or inhibitory effect (Fig. 1B). Additionally the effect of TMS intensity was tested by modulating the intensity of the given TMS between 35% and 95% of maximum stimulator output.

In the different single pulse TMS experiments 44 healthy right-handed men aged between 20 and 30 (mean age 24.3 $\pm$ 1.9 yrs $\pm$ std.) took part overall. TMS was performed using a Magstim 200 stimulator with a maximum output of 2,0 Tesla (Magstim Co., UK). A figure of eight magnetic coil (dual 70 mm coil) was used for focal stimulation. The coil was positioned tangentially to the skull with a handle orientation parallel to the sagittal axis.

For each subject the optimal scalp location for induction of motor evoked potentials in the contralateral biceps brachii muscle was identified. Single pulse TMS was then applied at decreasing intensities to determine motor threshold, following the guidelines established by the International Federation of Clinical Neurophysiology [34]. For Wernicke's area stimulation CP 5 in the International 10–20 System was chosen. This site correlates fairly well with the location of Wernicke's area [14,17]. In a subgroup CP5 location over the posterior temporal cortex was additionally verified using a commercially available MRI-based neuronavigation system (Fig. 2).

To measure the influence of TMS on picture naming latencies, magnetic stimuli were given to the motor cortex or to Wernicke's area at systematically varied time points prior to and post picture onset. The time interval from picture onset to stimulation was defined as the interstimulus interval (ISI), with negative values of ISI signifying that TMS preceded picture presentation. Since naming latencies decrease with repeated presentation of the same pictures [22] a new group of age-matched subjects was deployed for each part of the experiment. Intensity of TMS was set at 10% above motor threshold (MT) for evoking an EMG response in the biceps brachii muscle (between 55 and 65% of maximum output). Since it has been shown that MT is not related to the excitability of non-motor cortical areas [4] intensity of magnetic stimulation was set arbitrarily at 55% of stimulator output for stimulation of Wernicke's area.

To exclude that the discharge of the magnetic stimulator influences naming latencies in the present paradigm the potential facilitatory effects of the discharge click alone was explored. In these experiments the magnetic stimulator was discharged with the coil held perpendicular to the skull surface in order to prevent effective brain stimulation ("click alone" condition). The effects of the click alone on naming latencies were compared to TMS over the motor cortex or Wernicke's area (for details see [41]).

Single-pulse TMS was applied to the left motor cortex at the following time points in different groups of subject: ISI: $-3000$, $-1000$, $-500$, $-100$, $-50$, 0, $+100$, $+200$, $+300$ ms; baseline condition: naming without TMS (Fig. 1B). The detailed description of the experiments is given in [41]. Wernicke's area was stimulated at the following time bins: ISI –5000 $-3000$, $-1000$, $-500$ $-100$, $-50$, 0, $+100$, $+200$, $+300$ ms baseline condition: naming without TMS and sham stimulation. Additionally at –500 ms TMS intensity was varied (35%, 55%, 75%, 95%).

### *4.1. Results*

Accuracy of naming was unaffected by TMS over any of the stimulated areas. Approximately 5% of the

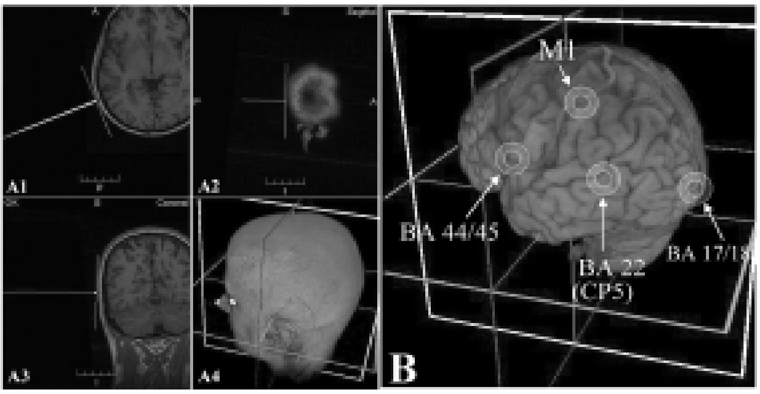

Fig. 2. Using a commercially available frameless stereotactic systems anatomical locations of CP5 and CP6, as well as the intermediate position between F5 and F7, were verified in a small subgroup of subjects to be over the posterior third of the superior temporal cortex (CP5, 6) or the inferior frontal cortex respectively.

items had to be excluded. Neither preceding nor consecutive TMS over the motor cortex with respect to picture onset revealed a significant influence on picture naming latencies. Single-pulse TMS over Wernicke's area resulted in a significant reduction of naming latencies with rTMS given at negative ISIs of 500 and 1000 ms (Fig. 3). Higher intensities (75%, 95%) had no effect. The TMS influence on naming latencies was specific to the dominant hemisphere since TMS of the Wernicke's homologue area of the right hemisphere had no impact on naming latency (detailed results are presented in [41]).

## 5. Influence of repetitive TMS on picture naming

The experiments using rTMS were build up on the previously demonstrated results. In these experiments trains of rTMS were applied at high or low frequencies, respectively (Fig. 1C). Additionally stimulus intensity was varied. We intended to investigate whether rTMS is capable of modulating language networks. Based on the finding of longer-lasting effects following motor or visual cortex stimulation [2,5] we postulated that rTMS over language related cortical areas may result in a more prolonged facilitatory effect on subsequent language function.

36 healthy men aged between 22 and 38 years (mean age 28.6 $\pm$ 3.2) participated overall in the rTMS experiments [23,38]. To study the influence of rTMS on picture naming latencies, stimulation was applied over either Wernicke's area ($n = 10$; BA22), the right hemisphere homologue of Wernicke's area ($n = 10$; BA22), Broca's area ($n = 10$; BA44) or the primary visual cortex ($n = 6$; BA17/18) as a control side. rTMS was applied using a Magstim Rapid stimulator (Magstim Co., UK). Since motor cortex stimulation in the previous single-pulse TMS experiments had no effect on naming latencies this site was omitted in the last parts of our studies.

rTMS trains with a frequency of 20 Hz and a duration of 2 s were delivered with a focal figure-of-eight magnetic coil (dual 70 mm coil). Since threshold is difficult to determine for a cortical areas outside the primary motor cortex as stated before, we again decided to use with rTMS a fixed stimulation intensity of 55% of the maximal output [4]. The coil was positioned tangentially to the skull, with the handle parallel to the sagittal axis and pointing occipitally. For the stimulation of Wernicke's area (W1, W2) – i.e. Brodmann area 22 or in anatomical terms the posterior third of the superior temporal gyrus – the coil was centered over CP5 of the international 10–20 system (Fig. 2). The homologous area of the right hemisphere (R1, R2) was stimulated over CP6. For Broca's area stimulation (B1, B2) – i.e. Brodmann area 44 or in anatomical terms frontal operculum of the inferior frontal gyrus – a site intermediate between F5 and F7 of the international 10–20 System was chosen. In an earlier study a disruption of ongoing speech processes was described after stimulation of this site [26]. The primary visual cortex (V1, V2) was stimulated at the occiput [1]. Compared to the discharge click of single-pulse TMS the discharge of the rapid stimulator is less loud. The possibility however remains, that the rTMS train acts as a warning stimulus, and influences the naming latencies. The potential facilitatory effects of the discharge clicks without concomitant TMS was therefore explored separately in 13 individuals [23]. The magnetic stimulator was discharged with the coil held perpendicular to

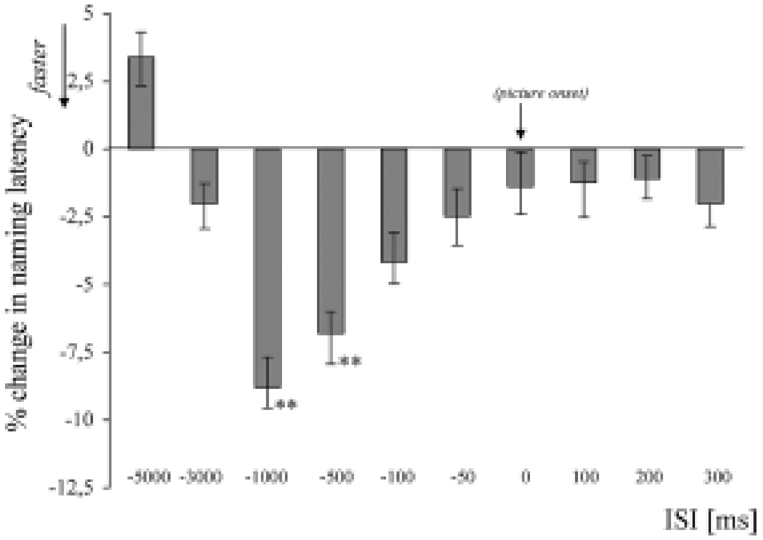

Fig. 3. Pooled results of all single-pulse TMS experiments [41] are shown. Interestingly a general unspecific reduction of naming latency was seen independent of the ISI, however a significant reduction ($p < 0.05$) of naming latencies was observed only if TMS preceded picture naming by 500 or 1000 ms.

the skull surface in order to prevent brain stimulation (sham stimulation: S1, S2).

To study the effect of stimulation intensity on the observed facilitatory effect seen in the first part of the rTMS studies with 55% of maximum stimulator output two additional stimulation intensities at 45% and 35%, respectively, were applied. Trains of 40 stimuli with a duration of 2 s were applied over Wernicke's area.

Finally the effect of low-frequency (1-Hz) rTMS on picture naming was investigated. In order to keep the number of stimuli in a single train constant compared to the high frequency trains, rTMS was carried out with a frequency of 1-Hz and with a train duration of 40 s at 55%, 45% or 35%, respectively, of the stimulator's maximal output.

Previous investigations have demonstrated that the naming latency of an object decreases when the object is shown several times [22]. The naming of the selected pictures in the rTMS experiments was therefore overtrained by presenting each picture eight times prior to the experiments.

The total set of stimulus pictures had 20 items containing two copies of each of the 10 selected pictures. The order of items was systematically varied. During each experimental task the complete set of 20 pictures was presented. The pictures were presented immediately (W1, B1, R1, V1 or S1) and two minutes after (W2, B2, R2, V2 or S2) the rTMS train had been completed [23,38].

### *5.1. Results*

Accuracy of naming was unaffected by rTMS over any of the stimulated areas. Approximately 5% of the items had to be excluded. There was no significant difference between the number of responses which had to be excluded across the different conditions among all subjects due to errors or tip of the tongue phenomena. The variability of naming latencies per condition was lower than in the single-pulse TMS studies [41]. This was expected given the selection of the stimulus pictures and the extensive pretraining.

rTMS over Wernicke's area led to a significant reduction in naming latencies immediately after stimulation (Fig. 4). Moreover, there was a significant gradual decline of this effect over the first block (Fig. 5). In the second block of pictures presented 2 min after the end of the rTMS train no facilitatory effect was seen (detailed presentation of the results in [23]). Naming accuracy was again not affected. Stimulation of Broca's area, the visual cortex or the Wernicke's homologue had neither an effect on naming latency, nor resulted in an increased number of errors. In contrast to the results of rTMS at 55% intensity, the statistical analysis of the measured naming latencies after rTMS with an intensity of 45% and 35% of maximal output did not reveal significant differences (Fig. 6) for any stimulation condition compared to baseline [38]. Low frequency (1 Hz) rTMS with the given stimulation parameters revealed no evidence of either an excitatory, or an inhibitory effect on picture naming latency [38].

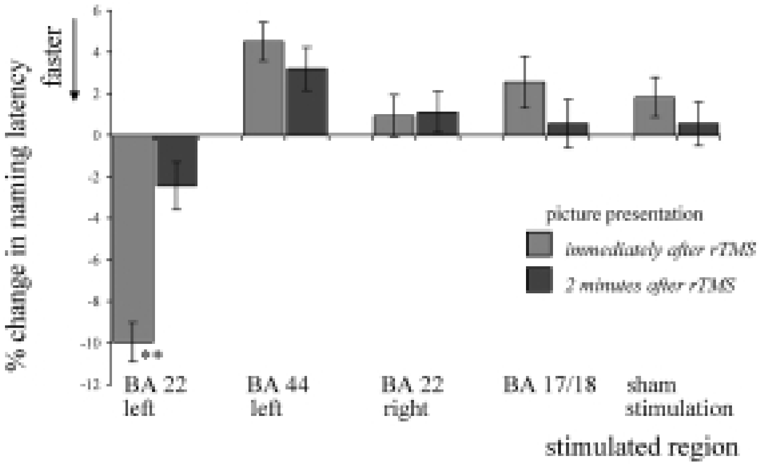

Fig. 4. Results of high-frequency rTMS to different targeted brain regions on picture naming latency. Latency is depicted as percent change relative to baseline (i.e., non-TMS) naming latency [23,38]. Only high-frequency rTMS over Wernicke's area (BA 22 left) preceding picture presentation showed a significant effect. Broca's area stimulation (BA44), visual cortex (BA 17/18) and right hemispheric homologue to Wernicke's area (BA 22 right) had no impact on naming latencies.

## 6. Discussion

A significant shortening of picture naming latencies was observed after single-pulse TMS over Wernicke's area. The accuracy of the response was not affected by this speed effect. After TMS over the dominant motor cortex or over the non-dominant temporal lobe, however, no facilitation of picture naming was observed.

In the rTMS experiments only rTMS of Wernicke's area had an impact on picture naming latencies resulting in a shortening of naming latencies without affecting the accuracy of the response. This was only detected immediately after rTMS over Wernicke's area. This effect subsequently decreased in strength, but remained significant for the whole group of pictures presented within 0.5 min following stimulation. After 2 minutes this facilitatory effect vanished.

All presented experiments showed a specific effect of either rTMS or TMS over Wernicke's area on naming latency while stimulation of all other areas had no such effect. For all experiments it has to be taken into account that we did not initially confirm the site of stimulation by the use of other methods such as magnetic resonance imaging combined with a frameless stereotactic system. However we subsequently verified (Fig. 2) that CP5 correlates well with the potential location of Wernicke's area in the posterior temporal lobe by means of MRI-based neuronavigation. The observed effect might nevertheless still be explained by an inadvertent activation of other cortical areas such as the inferior parietal cortex, or the sensory cortex. Additionally, there is yet no precise knowledge about the spatial resolution of rTMS and therefore, it is difficult to translate the observed functional effects into exact anatomical localizations. Mapping studies within the motor or visual cortex using single pulse TMS have demonstrated a spatial resolution of around 0.5 to 1 cm at the scalp surface (for review see [16,43]). During a prolonged train of rTMS, spread of the effects along cortico-cortical connections may well lead to less focal effects [27]. Furthermore, several studies using combinations of TMS and different neuroimaging methods [11,15,29,30] have demonstrated that in addition to local effects in the directly targeted cortex, TMS can exert transsynaptic effects onto functionally connected brain regions. Whether such transsynaptic distant effects are behaviorally relevant is unclear. Nevertheless, rTMS might affect an undeterminable volume in which the effects of the rTMS tail off (similar to a Gaussian distribution) over an area larger than the spatial resolution at the scalp surface.

The facilitatory effect appeared to be specific for stimulation of Wernicke's area as no changes in naming latencies were found following stimulation of Broca's area, the right hemisphere homologues to Wernicke's area, or the primary visual cortex. Furthermore, we were able to exclude the possibility that TMS or rTMS might function as a warning stimulus leading to an increase of attention and thereby resulting in a shorter reaction time by showing that the noise produced by the stimulator (sham stimulation) had no impact on naming latencies.

Facilitatory effects on simple reaction times might be explained by intersensory facilitation [39]. The phe-

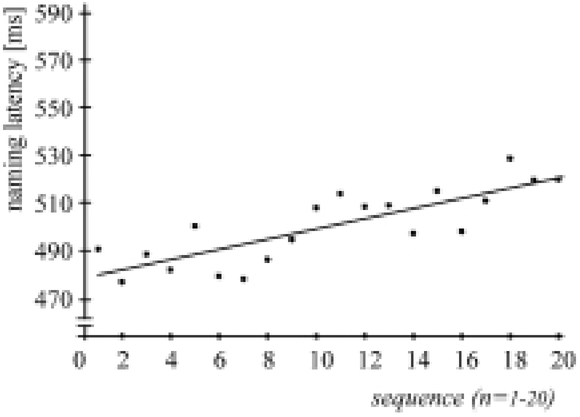

Fig. 5. The observed facilitatory effect on naming latency [23] vanished in a gradual manner over the first 30 s after the end of the high-frequency train ($p < 0.05$).

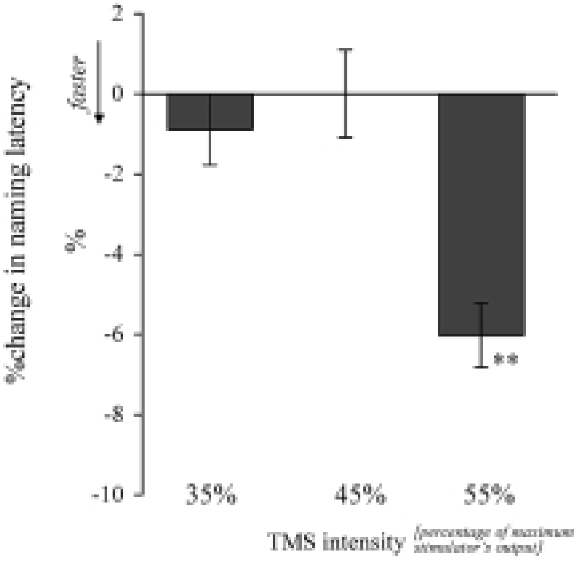

Fig. 6. The observed rTMS effect on naming is intensity dependent [38].

nomenon of intersensory facilitation describes the fact that simple reaction times can be shortened if the cue signal is accompanied by a second stimulus of various modalities at a maximal interstimulus interval of 50 ms [24]. In the present studies, intersensory facilitation cannot explain the observed facilitation of picture naming since in the present experiments significant facilitation was only found when TMS preceded picture presentation by at least 500 ms. Furthermore, intersensory facilitation increases with the intensity of the second signal. Therefore an increase of the facilitatory effect should be expected with increasing stimulus intensity of TMS. With rTMS this was true for the studied intensities, however for single pulse TMS the opposite, was observed for higher intensities (75 and 95% of stimulator output) [41]. Since intersensory facilitation is not modality-specific, the differential effects of TMS over Wernicke's area and the motor cortex cannot be explained by this hypothesis. A "non-TMS specific" increase in the subject's ability to react to a given stimulus is therefore an unlikely explanation of the observed facilitation of picture naming latencies after TMS over Wernicke's area.

The theory that neuronal networks rather than circumscribed cortical centers underlie cognitive function is nowadays widely accepted. In this regard we believe that TMS and rTMS have the potential to facilitate the neuronal network for language. Our results suggest that focal TMS leads to a lasting facilitation of local neuronal networks resulting in a general preactivation of consequent cortical activities. Since TMS leads to a simultaneous depolarization of a rather large population of neurons and interneurons, respectively, we postulate that these neurons might be rectified to certain extent. This alignment may result in a more time efficient processing within the local sensory language module. rTMS has the potential to synchronize neuronal activity [31]. Thus, an explanation for the observed rTMS effects on naming could be a comparable synchronization in the neuronal language network leading to a more efficient processing and throughput.

Since magnetic stimulation had an effect only following stimulation of Wernicke's area magnetic stimulation seems to be capable of inducing a facilitation only at the point of lexical search in the proposed neuronal network for speech generation. The processing of information in these neurons might be more effective when they are firing in unison. On the other hand neurons in Broca's area are not benefitted by this kind of facilitation as the motor program for speech generation is probably highly optimized already in adults.
